# New and old tools to evaluate new antimicrobial peptides

**DOI:** 10.3934/microbiol.2018.3.522

**Published:** 2018-06-29

**Authors:** Hector Rudilla, Alexandra Merlos, Eulàlia Sans-Serramitjana, Ester Fusté, Josep M. Sierra, Antonio Zalacaín, Teresa Vinuesa, Miguel Viñas

**Affiliations:** 1 Department of Pathology & Experimental therapeutics, Faculty of Medicine & Health Sciences, University of Barcelona, Feixa Llarga s/n 08907 Hospitalet, Barcelona, Spain; 2 Department of Clinical Sciences, Faculty of Medicine & Health Sciences, University of Barcelona, Feixa Llarga s/n 08907 Hospitalet, Barcelona, Spain

**Keywords:** antimicrobial peptides, bacteria, protozoan, fungi, atomic force microscopy, confocal microscopy, growth curves, death kinetics

## Abstract

The emergence of antimicrobial resistance due to the overuse of antimicrobials together with the existence of naturally untreatable infections well demonstrates the need for new instruments to fight microbes. Antimicrobial peptides (AMPs) are a promising family of molecules in this regard, because they abundantly occur in nature and the results of preliminary studies of their clinical potential have been encouraging. However, further progress will benefit from the standardization of research methods to assess the antimicrobial properties of AMPs. Here we review the diverse methods used to study the antimicrobial power of AMPs and recommend a pathway to explore new molecules. The use of new methodologies to quantitatively evaluate the physical effect on bacterial biofilms such as force spectroscopy and surface cell damage evaluation, constitute novel approaches to study new AMPs.

## 1. Introduction

Bacteria are the leading cause of infections worldwide but they affect individuals in developed and third world countries in different ways. In the latter, most bacterial infections are those known as classical, whereas in developed areas hospitalized patients or individuals who have undergone medical treatments, such as surgery, solid organ transplantation, and anticancer treatments, are the most vulnerable. In recent years, the progressive increase in the incidence of multidrug-resistant bacterial infections has raised concern. In 2014, the office of the President Obama published a detailed report on antimicrobial resistance, leading to the publication, on September 18, 2014, of an Executive Order entitled “Combating Antibiotic-Resistant Bacteria” (https://obamawhitehouse.archives.gov/the-press-office/2014/09/18/executive-order-combating-antibiotic-resistant-bacteria). This document included sections on new policies, changes in funding, and recommendations, among others. Following the election of Donald Trump, more than 30 scientific and social alliances and societies signed a document in which they expressed the hope these investments would be maintained and even expanded. Also in 2014, the World Health Organization (WHO) published, an extended report calling attention to this crucial health problem (www.who.int/drugresistance/documents/surveillancereport/en/). Bacterial resistance to antimicrobials has likewise attracted the attention of the governments of several other countries.

However, antibiotics research had an erratic history. The clinical use of antimicrobials started approximately in 1932, with the release of Prontosil (an antibacterial drug discovered at Bayer Laboratories, Germany), a molecule with a lethal effect on gram-positive cocci. Prontosil was the first sulphonamide and it ushered in the antibiotic era. Thereafter, new antibiotics were rapidly discovered, including penicillin and streptomycin, and became available for clinical use.

Recently, due mostly to economic, rather than to medical or scientific reasons, the pipeline of novel antimicrobial molecules under development has mostly closed [1]. Instead, most of the drugs under development are improved derivatives of those already on the market. This has had several non-negligible consequences. Firstly, modified compounds, while frequently enlarging the spectrum of drug activity or enhancing its antimicrobial effect, do not change the target, including the resistance mechanisms promoted in organisms exposed to these agents. New molecules, acting through newly recognized mechanisms of action and on different targets, are expected to be much more effective, but very few have been developed over the past several years. The recent emergence of new mechanisms of resistance and the scarcity of novel antimicrobial products able to target them account for the current growing concern and the revival of research efforts.

The main sources of the thousands of antibiotics discovered during the golden era of antibiotics were soil bacteria and fungi. In fact, antibiotics were long regarded as defense mechanisms of soil microbes, although this function has yet to be definitively demonstrated in nature [2]. Although the further exploration of natural products for their antibiotic activities is expensive and the chance of successes limited, the identification of not previously appreciated delivery methods or products, including those derived from natural molecules, will open up new research perspectives regarding antimicrobials [3]–[7].

Once a candidate molecule has been synthesized and purified, its properties, activities, and efficacy, but also its toxicity, must be investigated at the biological level. Clearly, a drug should exhibit greater toxicity towards microorganisms than on its human hosts. Moreover, not only bacteria but also fungi and protozoa produce infections. Therefore, in the process of exploring the antimicrobial action of a new molecule, the possible effect on other infectious microbes should be investigated as well.

This review examines the main methods used to investigate new antimicrobials and, in the form of a flow diagram presented at the end, describes the main criteria for their development.

## 2. Minimum inhibitory concentration

The minimum inhibitory concentration (MIC) is a parameter widely used to assess the susceptibility of microbes. It is defined as the lowest antimicrobial drug concentration that prevents visible growth of the microorganism after an overnight incubation. An advantage of the MIC is that it is quantitative and, if standardized procedures are used, the values obtained by different laboratories can be compared. Moreover, national agencies, mainly the USA's Clinical and Laboratory Standards Institute (CLSI) and the European Committee on Antimicrobial Susceptibility Testing (EUCAST) have published cut-off points based on criteria that are relatively homogeneous worldwide.

**Figure 1.**
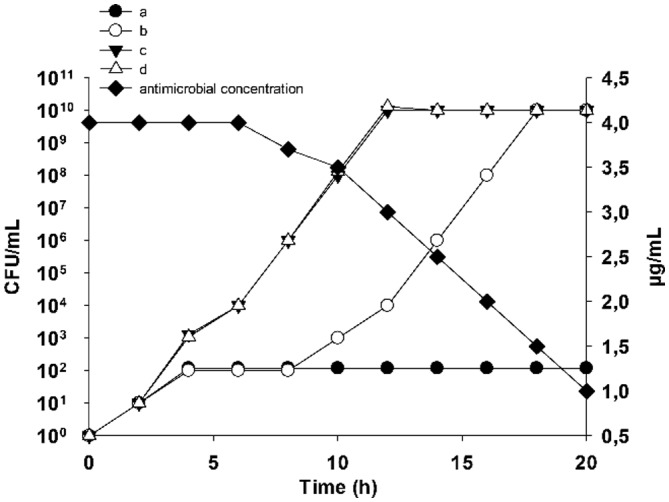
Comparison of the growth curves of different bacteria in the presence of an antibacterial agent added at 4 h. The growth kinetics of a control culture (open triangles), a resistant microbe (filled triangles), a moderately susceptible microbe (open circles), and a fully susceptible microbe (filled circles) are shown. Filled squares represent antimicrobial concentration.

**Figure 2.**
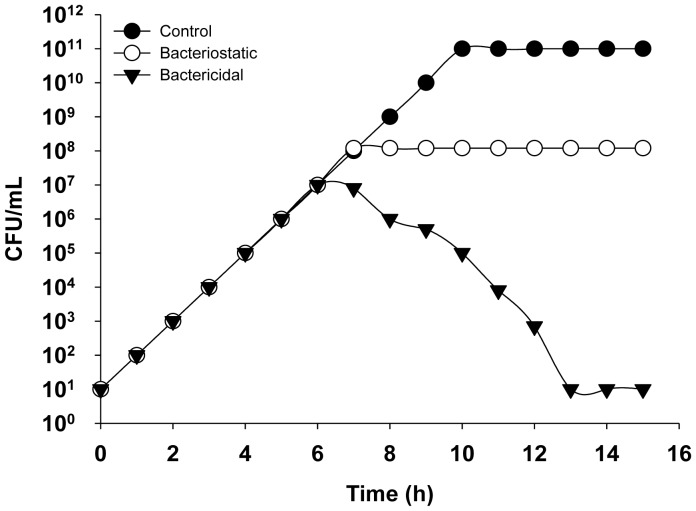
Growth kinetics of a bacterium in the presence of a bacteriostatic (open circles) and a bactericidal (filled triangles) agent.

Either solid (incorporating antimicrobials into the agar medium) or liquid medium can be used to determine MIC values. Liquid medium tests are conducted using either the microdilution (96-well microtiter plates) or the macrodilution (test tubes) method. However, while an excellent parameter for clinical purposes, the MIC has serious limitations in research. First, the inoculum density strongly influences the MIC value. Second, the definition of the MIC is vague, as, for example, neither “visible growth” nor “overnight” (typically 18 h) is well-defined. Moreover, visible growth is determined with the naked eye, and the MIC is a fixed value determined after 18 h of incubation. However, while the presence of visible growth after 18 h should be interpreted as a lack of antimicrobial action, this may not precisely be the case, as illustrated by a simple theoretical example: Microbe A is susceptible to 4 µg of drug X/mL, and a fully resistant isolate (B) is resistant to 200 µg of drug X/mL. After incubation of microbes A and B in medium containing 4 µg X/mL, the growth was visible in both tubes (Figure 1), but the history of the cultures is completely different. Whereas the growth of culture B started immediately, as in the control experiment, culture A was inhibited for the first 12 h, during which time the antibiotic concentration was >3 µg/mL, but began to grow thereafter. This is frequently referred to as “regrowth” and is discussed controversially, although its existence demonstrates the weakness of MIC as a parameter. Moreover, antimicrobial action may be bactericidal, in which the peptide kills the bacterium, or bacteriostatic, in which bacterial growth is completely inhibited (Figure 2). These two mechanisms can be distinguished by plotting a growth curve of the bacterium in the presence and absence of antimicrobials.

## 3. Minimal biocidal concentration

A much more easily defined and informative parameter is the minimal biocidal concentration (MBC): the lowest concentration of an antimicrobial agent required to kill a particular bacterium. Bacterial killing is confirmed when the test culture contains no growing cells. However, the MBC also depends on the chosen methodology. For instance, growth is a function not only of the experimental conditions but also the metabolic state of the bacterium. Thus, bacteria may be viable but non-cultivable (VBNC) due to their poor metabolic activity. Nonetheless, while unable to divide, they are still alive because under the appropriate conditions allowing their “resuscitation” they are “re-cultivable”. VBNC unable to grow in standard medium are smaller and have lower levels of nutrient transport, ATP production, and macromolecular biosynthesis but they may still be able to survive for as long as one year [8]. Clinically, VBNC may be the source of recurrent infections but they are not taken into account in MBC determinations. Furthermore, experimental MBC values are often beyond the limits that are pharmacologically achievable in a therapeutic setting; thus, the MBC is mostly valuable in theoretical studies, not in clinical microbiology. Furthermore, whether an antimicrobial is biostatic or biocidal in most cases strongly depends on its concentration.

## 4. Growth curves

In the search for new antimicrobials, an understanding of the dynamic interactions between the putative drug and the microorganism is clearly important. The activities of natural and synthetic peptides can be easily followed by plotting a growth curve, that is, a graphical representation of the growth of the bacterium of interest in a freshly inoculated culture. During the exponential phase, growth proceeds at a maximal rate (µmax), which depends upon the characteristics of the bacterium and the environmental conditions, including temperature, oxygen availability (for some bacteria), light, medium composition, etc. The growth rate (µ) can be calculated as N_t_ = N_0_ × e^µt^ and thus as µ = (log_n_N_t_ − log_n_N_0_)/t, where N_t_ is the number of individuals at time t and N_0_ the number of individuals at time 0. The exponential phase of growth is followed by a transition phase in which µ decreases until it reaches 0, marking the stationary phase. For a given microbe cultured under standardized conditions, the growth curve is highly reproducible whereas the addition of an antimicrobial disrupts the growth curve. When added during the exponential phase, antimicrobials can be evaluated as bacteriostatic or bactericidal (Figure 2). Although, in principle, the desired effect of an antimicrobial is bactericidal rather than bacteriostatic, this is not universally true. Many bacteriostatic antimicrobials are excellent therapeutic agents because they promote host defense mechanisms, which help to eradicate the bacterium. Furthermore, bacteriostatic agents can also be used to prevent nosocomial infections as well as infections in indwelling medical devices.

## 5. Death kinetics

The best way to accurately determine the pharmacodynamics of drug is to plot a so-called time-kill curve. The drug under study is added to a starting bacterial inoculum of 10^6^ CFU/mL in MHBCA (Muller-Hinton broth cation adjusted) medium, and parallel experiments adding 25% mammal serum; at different times, aliquots are retrieved aseptically for bacterial counting.

**Figure 3.**
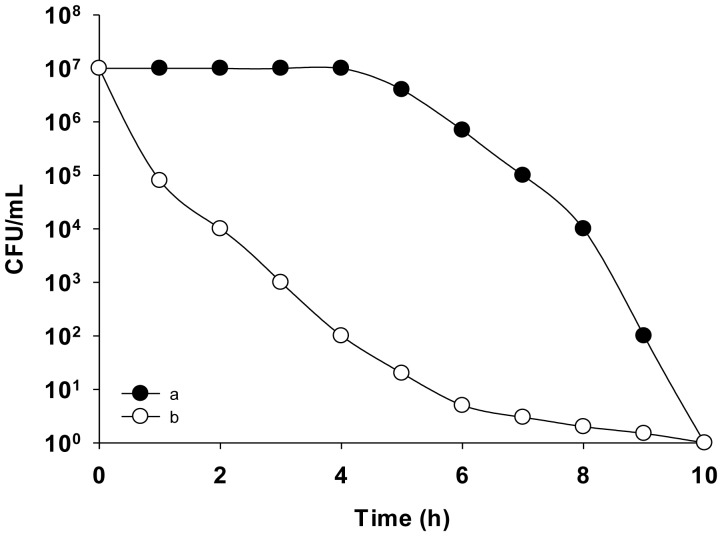
Death kinetics of two antimicrobials: one slow-acting (open circles) and the other fast-acting (filled circles).

The advantage of a time-kill curve is that provides a dynamic picture and thereby avoids the limitations of fixed time-point studies (Figure 3). Information on the behavior of a drug during its first hours of bacterial contact is extremely important, if only the initial and final time-points are analyzed, the different intervening processes will be missed, as described for the MIC.

**Figure 4.**
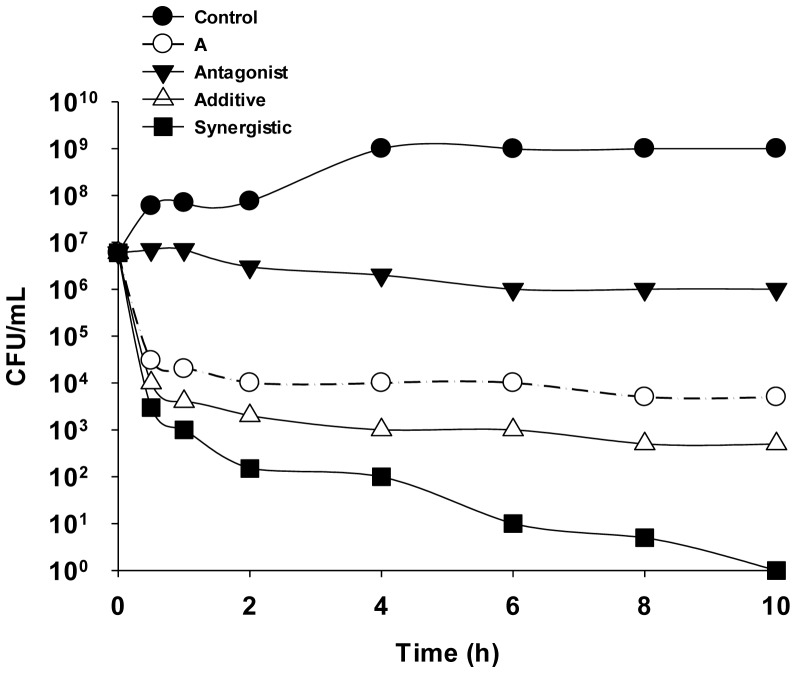
Comparison of the time-kill curves of bacteria incubated in the presence of various antimicrobials (A means the antimicrobial alone).

Death kinetics can also be used to test for possible synergies between two or more compounds. Synergism occurs when a drug combination results in a reduction of the bacterial counts by two or more logarithms (decimal) compared to the most active drug alone. A reduction of less than two logarithms indicates an additive relationship. A null logarithmic reduction is called indifference, whereas antagonistic effects are reflected by an increase of the bacterial counts by two or more logarithms compared to the counts obtained with each drug separately (Figure 4). In many cases, these data reinforce those obtained by checkerboard (see Section 6).

## 6. Antimicrobial peptide interactions

**Figure 5.**
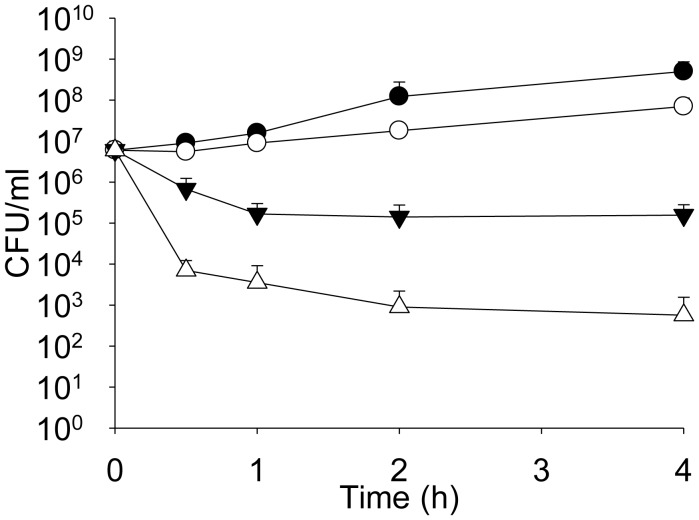
Effect of the control (filled circles), 4 µg imipenem (IMI)/mL (open circles), 8 µg AMP38/mL (filled triangles), and 8 µg AMP38/mL + 4 µg IMI/mL (open triangles) on *Pseudomonas aeruginosa* strain PA116136 [9].

The interaction between peptides and other antimicrobials, or between two antimicrobial peptides (AMPs) can be determined quantitatively from the fractional inhibitory concentration (FIC) using the checkerboard technique. The FIC of drug A can be calculated as: (FIC A) = (MIC of drug A in combination)/(MIC of A), and the FIC of drug B as: (FIC B) = (MIC of drug B in combination)/(MIC of B). The FIC index (FICi) is calculated by adding FIC A and FIC B. FICi < 0.5 indicates a synergistic interaction, FICi values between 0.5 and 4 no interaction, and FICi > 4 an antagonistic interaction. Together with calculation of the FICi, determinations of death kinetics provide a useful tool to explore drug interactions whereas growth curves reveal the interactions between two or more antimicrobials. Figure 5 shows an example in which the synergistic effect of imipenem and a newly synthesized AMP (amp38) was determined [9].

In fact, AMPs have been described as synergistic agents with a wide variety of antimicrobials. The inhalation therapy of respiratory infections suffered by cystic fibrosis patients demonstrate strong antimicrobial synergy of Polymyxin B in combination with silver nanoparticles [10]. Moreover, in fungal infections treatment, AMPs are also regarded as candidates to act through synergistic effect with already known antifungals. A good example is Ctn (15–34), a carboxyl-terminal fragment of crotalicidin (a cathelicidin from the venom gland of a South American rattlesnake), the molecule has *per se* a certain antifungal activity, but an enhancement of antifungal properties was found when combined with amphotericin B [11]. Even looking at viruses different examples of synergism have been reported. It has been shown that synergy emerges when Env (a protein involved in HIV penetration) engages multiple co-receptors prior to inducing fusion and when high-affinity inhibitory peptides are present [12]. Finally, it is worthy to mention that such synergistic effects have been demonstrated even between AMPs and inorganic molecules. This is the case of carbon monoxide-releasing molecules (CORMs): a novel class of compounds (for example the light-activated metal complex [Mn(CO)3(tpa-κ3N)]Br) they have shown high synergism with some AMPs such as colistin [13]. This strongly suggests the need to explore interactions when exploring new AMPs, since even when antimicrobial activity may be weak, their use as enhancers of antimicrobial action of others may restitute their interest. One more question should be addressed when taking into account that, at least in one case, AMPs have been found to be potentially useful to eradicate biofilms and to increase susceptibility to already known antimicrobials [14].

## 7. Measuring the efficacy of AMPs against biofilms

Traditional antimicrobial susceptibility tests (from disk diffusion to broth microdilution methods) used to calculate the MIC and thus to define the susceptibility breakpoints predicting therapeutic success are performed mostly using planktonic bacteria. However, planktonic microbes are very infrequent in infections. Rather, most infectious diseases are caused by bacteria living in a biofilm, usually attached to a surface or an interface. Within the biofilm, the bacteria are embedded within a slimy extracellular matrix of bacterial origin and they adhere to each other [15]. Biofilm bacteria have a much greater resistance to antimicrobials than their planktonic counterparts [16] and the corresponding susceptibility breakpoints have not been established [17]. This difference in antimicrobial susceptibility between planktonic and biofilm populations of the same organism is due to differences in the diffusion of antimicrobials and to complex physiological changes in biofilm microbes [18],[19]. Thus, for biofilm infections, using the MIC to predict the success of antimicrobial treatment is often ineffective, such that susceptibility tests for biofilm-growing bacteria are needed. The most relevant parameters currently used to evaluate the *in vitro* antimicrobial activity of antimicrobial compounds in biofilm-growing bacteria are the minimal biofilm eradication concentration (MBEC) and the biofilm prevention concentration (BPC) [20]. The effect of antimicrobial compounds on the viability and physical stability of the biofilm can be studied with confocal laser scanning microscopy (CLSM) and atomic force microscopy (AFM), respectively [21],[22].

## 8. Minimal biofilm eradication concentration

Given the serious challenges associated with infections caused by biofilm microbes and the role of biofilms in promoting recurring infections [23], the development of accurate methods to evaluate the efficacy of new antimicrobial compounds against biofilms is crucial.

The MBEC is defined as the lowest concentration of antibiotic required to eradicate the biofilm [24], in other words, the lowest concentration preventing bacterial regrowth from the treated biofilm.

The MBEC assay uses the Calgary biofilm device (CBD), a 96-well plate with pegs or projections built into the lid [24]. Each peg provides a surface for bacterial adhesion, colonization, and the formation of a uniform biofilm. The lid is used in conjunction with special troughs for growing, washing, and incubating the bacteria. The growing microorganism are cultured in an opportune growth medium and allowed to form biofilms on the pegs for 4 to 24 h (depending on the specific bacterial growth rate). Bacterial motility greatly influences biofilm formation on the pegs, such that more motile microorganisms, which have a greater tendency to aggregate, form robust biofilms on the pegs [25]. For example, microorganisms such as *Pseudomonas* *aeruginosa* and *Escherichia* *coli* are flagellated and motile and form high-density biofilms on CBD pegs [26],[27] whereas non-motile *Staphylococcus* *aureus* is much less effective in biofilm formation [28].

Once the biofilms are formed, the pegs are rinsed and placed onto flat-bottom microtiter plates, where they are incubated for 18–20 h at 37 °C in the presence of different concentrations of antimicrobials. Then, the pegs are again rinsed and transferred to antimicrobial-free medium in a biofilm recovery plate. Gentle centrifugation (805 g for 20 min) or a 5-min sonication at room temperature is used to transfer biofilms from the pegs to the plate. The MBEC values are then determined spectrophotometrically, by measuring the optical density at 620 nm. This method permits incubation of the biofilm with antimicrobials at different time-points, with daily rinsing and antimicrobial renewal [29].

It is important to point out that the minimal concentration of an antimicrobial required to eradicate a biofilm (MBEC) may be higher than the MIC determined against planktonic bacteria of the same species or isolate [3],[9].

## 9. Biofilm prevention concentration

Considerable efforts have also been devoted to prevent biofilm formation during the early stages of the colonization process [30],[31], by effectively eradicating bacteria with the appropriate antimicrobial therapy [32]. The biofilm prevention concentration (BPC), defined as the antibiotic concentration required to prevent biofilm formation during the early stages of colonization, can be used to correlate *in vitro* measurements with the therapeutic results and may be a better indicator than other concentration-dependent parameters [21].

A modification of the MBEC assay can be applied to determine BPC values. A planktonic bacterial suspension is incubated in the CBD plates in growth medium containing different concentrations of antimicrobials [33]. After 4–24 h (depending on the specific bacterial growth rate) incubation, the pegs are rinsed, placed in antimicrobial-free medium in a biofilm recovery plate, and sonicated for 5 min. The detached bacteria are incubated for 4–24 h and the optical density (620 nm) is measured to determine the minimal concentration of antimicrobial preventing biofilm formation.

Its well-established equivalence or similarity with the MIC, demonstrates the utility of the BPC in assessing antimicrobial-mediated reductions in bacterial density to prevent biofilm formation. Use of the BPC could also improve treatment strategies aimed at eradicating biofilm-producing bacteria already during early biofilm formation rather than at the mature biofilm stage.

## 10. Determination of bacterial viability in biofilms using confocal laser scanning microscopy

**Figure 6.**
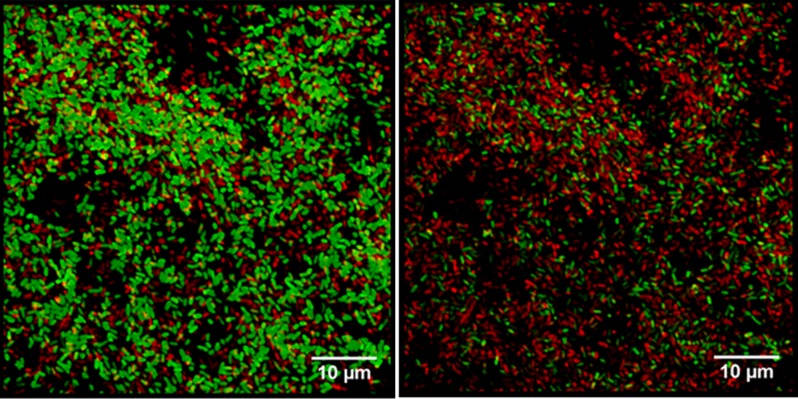
Confocal laser scanning microscopy images of *P. aeruginosa* biofilms stained using the LIVE/DEAD Bac Light bacterial viability kit. Green indicates viable bacteria, and red dead bacteria. (A) Most of the cells stain green, indicating a high level of bacterial viability. (B) Most of the cells are dead, indicated by their red fluorescence. Scale bar = 10 µm.

A biofilm is a complex and multicellular structure that harbors physiologically distinct subpopulations of bacteria that together from a community able to adapt to rapidly changing environmental conditions [34]. The efficacy of an antibiotic with respect to biofilm viability, both over time and across the different layers of the biofilm, can be studied using CLSM [35],[36]. In biofilms exposed to antimicrobial agents, CLSM reveals the effectiveness of the drug against the (metabolically active) outer layers and (metabolically attenuated) inner layers, and thus the time-dependent destruction of the biofilm. In an analysis of antimicrobial activity on *P. aeruginosa* biofilms, CLSM showed that some agents (such as ciprofloxacin) act preferentially on bacteria with high metabolic rates, whereas others (such as colistin) are effective only against bacteria with low rates of metabolism (Figure 6) [37],[38],[40].

To prepare the biofilm for CLSM, it is stained with a mixture of SYTO 9 and propidium iodide prepared at a dilution ratio of 1:2 (LIVE/DEAD Bac Light bacterial viability kit; Figure 6). Live bacteria stain green and dead bacteria red [41]. The CLSM images are then analyzed using ImageJ software (National Institutes of Health, Bethesda, MD, USA) [33] or other software tools.

## 11. Visualization of the effects of antimicrobials by atomic force microscopy

Invented over three decades ago [42], AFM not only reveals details of the cell surface, but also allows biological samples to be mechanically mapped, touched, dragged, pulled, pushed, or indented at the single molecular level. The resulting information provides valuable insights into the nanomechanical properties of living cells in their physiological environment and supports the information gained from other methodologies commonly used to assess antimicrobial activity, such as the MIC, minimal eradication assays, CLSM, and flow cytometry.

AMPs alter the physical properties of the cell, specifically, their morphology, volume, surface roughness, and stiffness [43],[44]. Data on roughness, obtained by software analysis, are complementary to viability assays. AFM analyses of the integrity of the cell surface and of membrane disruption can reveal whether treated cells display altered cell membrane characteristics, a loss of turgor, and a roughened surface, including bleb formation, with the eventual loss of the membrane permeability barrier and leakage of the intracellular content [45]. Imaging and the subsequent analysis of cell integrity have been used to evaluate alternative or complementary treatments.

**Figure 7.**
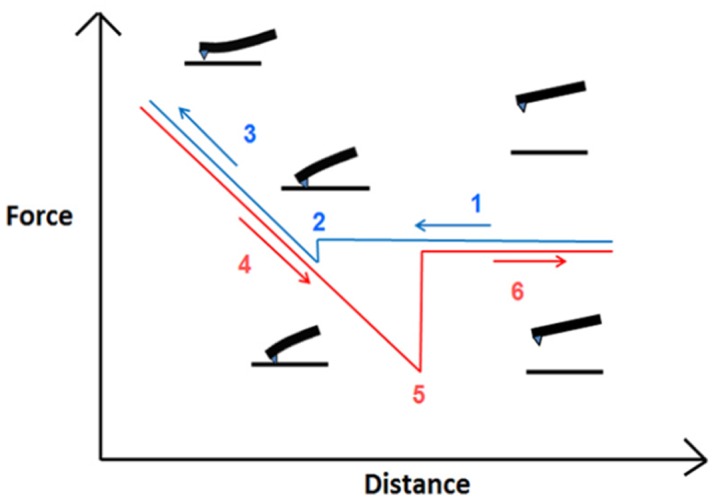
Schematic representation of the different phases in a force-distance curve obtained from AFM force spectroscopy. The blue line corresponds to the approach trace, and the red line to the retraction trace.

Gonçalves et al. [46] evaluated the antifungal activity of AMP Psd1, isolated from a defensin secreted by *Pisum sativum*, against three *Candida albicans* variants, one of them a mutant deficient in glucosylceramide synthase, conferring resistance to the peptide. AMP Psd1 significantly increased surface roughness, an indicator of relevant wall disorganization, resulting in a loss of stiffness of the treated cells of both the wild-type and the clinical isolate. Mularski et al. [47] used time-resolved AFM to study the pore-forming activity of the AMP Caerin 1.1 and its target. Adhesion forces, adhesion energy, the cell's Young modulus and other physic-mechanical parameters can be extracted using AFM-force spectroscopy, through force-distance (F/D) curves. These describe the deflection of a cantilever when approaching and contacting the sample and the displacement along the z axis (Figure 7).

**Figure 8.**
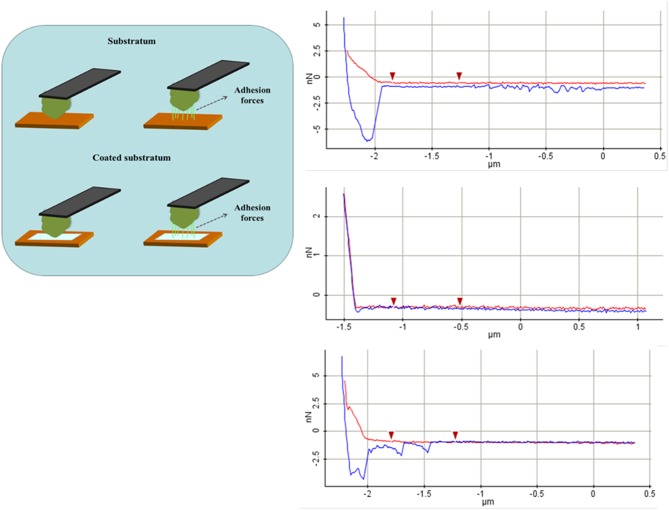
Representation of the retraction of the cantilever and the associated adhesion forces. As the cantilever moves away from the sample, the tip and surface molecules can detach in different ways. The adhesion force corresponds to the maximum external pulling force necessary for detachment to occur. On the right: Schematic representation of the different phases in a force-distance curve obtained from AFM force spectroscopy. The blue line corresponds to the approach trace, and the red line to the retraction trace.

As the cantilever approaches the cell (red trace in graphics), there are no changes in its deflection due to the absence of interaction forces at that point; however, when the tip reaches the sample, attractive forces such as Van der Waals forces, overcome the cantilever spring constant, forcing the tip to “jump to contact” the sample. Variation during the retraction regime (blue trace) is often characterized by a prolonged extension due to the tip remaining in contact with the sample, such that the cantilever is reflected downwards. A “jump” of the cantilever indicates its return to its final resting position. In terms of their nanomechanical properties, AMP-treated cells are less stiff than untreated cells [48], as shown by the final linear portion of the respective F/D curve. Moreover, the spring constant of the cell decreases in response to increasing AMP exposure, presumably due to the disintegration of the cell wall. Conversely, adhesion forces, generated between the tip and the cell surface, are considerably higher in AMP-treated cells. These forces represent the maximum external pulling force needed to separate the tip from the bacterial surface and the force needed to undo the recently formed bonds. Here again, degradation of the cell wall eases the penetration of the tip through the peptidoglycan layer and generates a higher number of contact points between them, finally increasing the force necessary to detach the cantilever from the cell [3]. As see in Figure 8, this force can either be continuous until detachment occurs or it can develop as several peaks, or jumps, of the pulling force towards zero when detachment is partial and bonds remain, a process referred to as sequential detachment. Finally, the adhesion energy is the sum of the amounts of energy needed to detach the AFM tip from the cell surface and to deform the cell close to its surface [49],[50]. It is calculated by integrating the area under the detachment force curve over the z displacement.

Pan et al. evaluated the interaction between the cell membrane and an AMP by studying the membrane lytic activity of a prion peptide (106–126). They reported decreases in both the Young modulus E of the bilayer and the bilayer puncture force, regardless of the cholesterol concentration of the supported lipid bilayers. In fact, a recent study by Henderson et al. showed that several AMPs reduce the edge stability of lipid membranes, thus altering the porosity of the cell membrane and causing the appearance of worm-like structures at high peptide concentrations. The specific interaction forces between AMPs and membrane components can be explored using single molecule force spectroscopy. This method was used by Oh et al., who characterized the nanoscale effects of the polycationic peptide polymyxin B (PMB) on bacterial membranes by determining the short-range interaction regime mediated by electrostatic forces between lipopolysaccharides and PMB, with 30 pN determined as the average unbinding force.

These examples demonstrate the versatility of AFM as a tool for biomedical research that provides information on many topographic and nanomechanical parameters of microbial and non-microbial cells.

## 12. Measuring the efficacy of antimicrobials against protozoa

Protozoan parasites include the well-known genera *Trypanosoma*, *Plasmodium*, and *Leishmania*. These organisms cause several serious human diseases that hamper the lives of people mostly in developing countries. Thus far, the control and treatment of protozoan diseases has depended on a rather small number of antiparasite drugs, which are frequently highly toxic and of low efficiency. Moreover, resistance of the parasites to these drugs is becoming increasingly common [51]. Thus, novel compounds and/or strategies are needed, including delivery systems [52] and new molecules, such as AMPs [53]. Membranes and DNA topoisomerases have attracted considerable interest as potential targets for novel antiparasitic therapeutics [54],[55].

With respect to AMPs, protozoa have received less attention than other microorganisms; however, they may be readily accessible targets because, except in the resistant cyst stage, they do not present structured external barriers, such as the bacterial capsule, the outer membrane of gram-negative bacteria, the thick peptidoglycan layer of gram-positive bacteria, or the compact fungal wall. The absence of these barriers allows direct interactions between the parasite and the AMP, which could facilitate an interpretation of the results. Nonetheless, in addition to determining the biocidal power and therapeutic index of an anti-protozoal therapeutic, its mechanism of action, lethal activity, and other effects, such as membrane permeation or modification of the energetic parameters of the parasite, must be elucidated to obtain valuable information on other potential targets as well as further drug development.

How AMPs perturb and destroy parasites can be studied using AFM, to examine morphological and structural damage of the cell surface, and transmission electron microscopy, to assess structural modifications to the parasite. The topographical relationship of the parasite with the AMP and the identity of intracellular targets, including the induction of apoptosis, can be investigated using confocal microscopy (see below). However, most protozoa have complex life cycles involving intracellular stages. Thus, as a first step, AMP activity should be assessed in extracellular parasites, before time-consuming and labor-intensive intracellular tests are performed.

Methods used to examine the efficacy of an AMP against a parasite include the following:

Cell proliferation measurements: As cell counting is tedious and subject to bias arising from reader expertise, fatigue, etc., the staining of viable cells using chromophores such as MTT, XTT, or resazurin allows an automated, colorimetric, and quantitative measurements of viable cells.

Cytotoxicity against intracellular parasites: The direct examination of mammalian cells stained with Giemsa's azur-eosin methylene blue reveals those infected with the parasite. A minimum count of 200 cells from different fields is required; the results are expressed as parasites per cell.

Assessment of plasma membrane permeation: The interaction between AMPs and the plasma membrane phospholipids of the target cells has been well-documented. For many AMPs, the peptide inserts into the membrane, disrupting its integrity and therefore its function as a permeation barrier, resulting in a lethal loss of internal homeostasis. For other AMPs, lethality is produced intracellularly, after their translocation across the membrane. Plasma membrane integrity can be assessed using fluorescence techniques, including cationic stains such as Syto9 or propidium iodide. Both of these vital dyes bind intracellular nucleic acids such that their entrance into the cell implies severe membrane damage, as also described in bacteria. Small or transient membrane damage results in plasma membrane depolarization, which can be followed using the sensitive probe bisoxonol, an anionic fluorescent molecule that reveals the discrete permeation of ions able to dissipate ion gradients. Mitochondrial membrane depolarization can be investigated based on the differential accumulation of rhodamine 123, a cationic fluorescence dye that enters metabolically active mitochondria.

Confocal microscopy: Using this technique, cells stained with different simultaneous labels can be examined and the intracellular target of a fluorescently labeled peptide identified. For example, organelles can be selectively stained and their distribution pattern compared with that of the tagged peptide. Among the fluorophores used for confocal microscopy are AMPs labeled with fluorescein, MitoTracker red (mitochondrial staining), and DAPI (nuclear and kinetoplast dye).

Measurement of the oxygen consumption rate: If oxidative phosphorylation, rather than glycolysis, is the main source of energy for the parasite, then dissolved oxygen, as an indicator of oxygen consumption, can be measured, typically using a Clark electrode or, as done traditionally, Warburg's instrument [56].

Microscopy. As in bacteria, the visualization of morphological alterations in parasites confirms antimicrobial action. Ultrastructural alterations of parasites can be viewed using transmission electron microscopy, and the three-dimensional surface morphology of the organisms using AFM. Sample treatment form AFM is minimal and the cells remain viable so that they can be evaluated physiologically in parallel.

Assessment of apoptosis by flow cytometry. Sublethal concentrations of peptides tend to cause apoptosis rather than necrosis, induced by higher doses. Apoptosis also occurs in response to slow permeation of the AMP or when the target is not the membrane but an intracellular organelle. Cell cycle analysis by flow cytometry, based on separation of the cells according to their DNA content, is a fast and easy method to study apoptosis. Ethanol-fixed, permeabilized protozoa are stained using propidium iodide and apoptotic cells then identified based on the appearance of characteristic peaks on the resulting histogram.

## 13. Evaluation of the antifungal activity of AMPs

Procedures to evaluate novel antifungal peptides are similar to those described above for antimicrobial peptides. However, as yeast and molds are eukaryotes, important differences involving nutritional requirements, optimal temperature, and duplication rate must be considered.

Standardized protocols for both yeasts and molds have been developed by the CLSI [57],[58] and EUCAST [59],[60]. In the case of the microdilution method, the main difference between the protocols of the two institutions involves the end point, based on the extended duplication time of these microorganisms. The CLSI use the same protocol developed for prokaryotic cells but with a 48-h incubation time, while in the EUCATS protocol results are obtained spectrophotometrically at 24 h. The MIC is defined as the lowest concentration resulting in a 50% (90% in some cases) inhibition of growth compared with the control.

Dynamic studies on the interaction between drugs and fungi should be performed as described for bacterial strains, albeit with differences in terms of the appropriate medium (RPMI), the inoculum size, and an extension of the incubation time up to 48 h.

The method to determine the antibiofilm activity of an antifungal agent differs from the bacterial protocol mainly in how the results are read. Similar to BPC and MBEC determinations for bacteria, the metabolic activity of fungi is measured using XTT or resarzurin [61] and the resulting tetrazolium compound monitored spectrophotometrically. However, for yeasts and molds, the BPC and MBEC are defined as the lowest concentration of the drug that yields a 90% reduction in metabolic activity vs. the untreated control.

Other techniques to study antifungal activity are being introduced. Of particular interest is flow cytometry [62]. The cells are stained with two different fluorescent dyes, one of which penetrates normal or intact cells, and the other only cells with a disrupted membrane, i.e., dead cells. Flow cytometry allows for single-cell fluorescence investigations. Hence, after the analysis of a suspension of cells treated with the antifungal peptide, a count of live and/or dead cells is obtained. The major advantages of this technique are the ability to study a large population of cells and the speed of the cell-by-cell analysis.

## 14. Remarks

**Figure 9.**
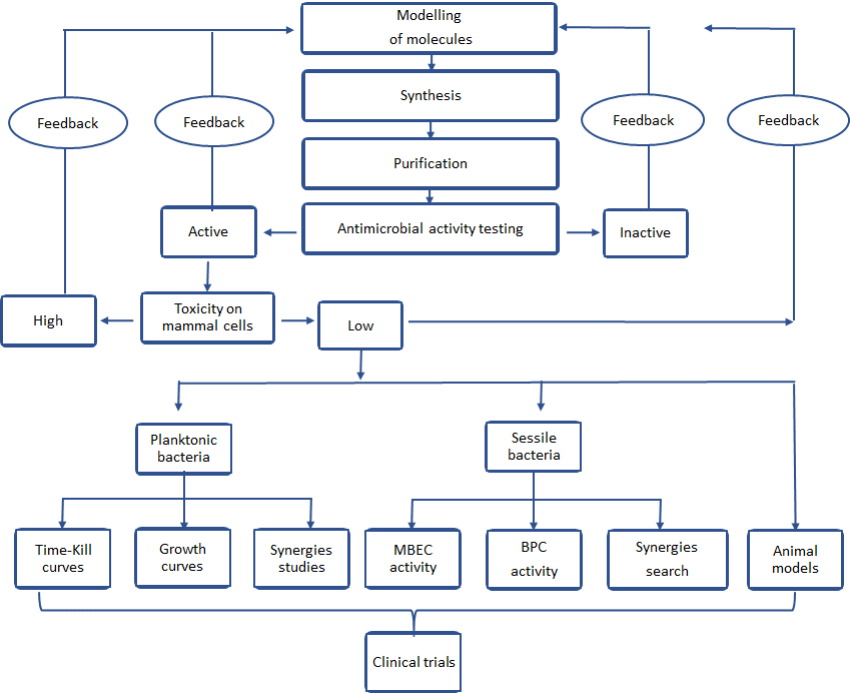
Sequence of techniques to be done at the initial search of new AMPs.

In summary the antimicrobial effect of a new molecule should be assessed in several steps, which are summarized in the flow chart shown in Figure 9.

## References

[1] Donadio S, Maffioli S, Monciardini P (2010). Sources of novel antibiotics—aside the common roads. Appl Microbiol Biotechnol.

[2] Sengupta S, Chattopadhyay MK, Grossart HP (2013). The multifaceted roles of antibiotics and antibiotic resistance in nature. Front Microbiol.

[3] Sans-Serramitjana E, Fusté E, Martínez-Garriga B (2016). Killing effect of nanoencapsulated colistin sulfate on *Pseudomonas aeruginosa* from cystic fibrosis patients. J Cyst Fibros.

[4] Bilal M, Rasheed T, Iqbal HMN (2017). Macromolecular agents with antimicrobial potentialities: A drive to combat antimicrobial resistance. Int J Biol Macromol.

[5] Rabanal F, Grau-Campistany A, Vila-Farrés X (2015). A bioinspired peptide scaffold with high antibiotic activity and low in vivo toxicity. Sci Rep.

[6] Townsend CA (2016). Convergent biosynthetic pathways to β-lactam antibiotics. Curr Opin Chem Biol.

[7] Azmi F, Skwarczynski M, Toth I (2016). Towards the development of synthetic antibiotics: designs inspired by natural antimicrobial peptides. Curr Med Chem.

[8] Oliver JD (2005). The viable but nonculturable state in bacteria. J Microbiol.

[9] Rudilla H, Fusté E, Cajal Y (2016). Synergistic antipseudomonal effects of synthetic peptide AMP38 and carbapenems. Molecules.

[10] Jasim R, Schneider EK, Han M (2017). A fresh shine on cystic fibrosis inhalation therapy: Antimicrobial synergy of Polymyxin B in combination with silver nanoparticles. J Biomed Nanotechnol.

[11] Cavalcante CSP, de Aguiar FLL, Fontenelle ROS (2018). Insights into the candidacidal mechanism of Ctn[15-34]—a carboxyl-terminal, crotalicidin-derived peptide related to cathelicidins. J Med Microbiol.

[12] Melikyan GB (2017). How entry inhibitors synergize to fight HIV. J Biol Chem.

[13] Betts J, Nagel C, Schatzschneider U (2017). Antimicrobial activity of carbon monoxide-releasing molecule [Mn(CO)3(tpa-κ3N)]Br versus multidrug-resistant isolates of Avian Pathogenic *Escherichia coli* and its synergy with colistin. PLoS One.

[14] Moon SH, Zhang X, Zheng G (2017). Novel linear lipopeptide paenipeptins with potential for eradicating biofilms and sensitizing Gram-negative bacteria to rifampicin and clarithromycin. J Med Chem.

[15] Hall-Stoodley L, Costerton JW, Stoodley P (2004). Bacterial biofilms: from the natural environment to infectious diseases. Nat Rev Microbiol.

[16] Davies D (2003). Understanding biofilm resistance to antibacterial agents. Nat Rev Drug Discov.

[17] Döring G, Flume P, Heijerman H (2012). Treatment of lung infection in patients with cystic fibrosis: current and future strategies. J Cyst Fibros.

[18] Stewart PS (1996). Theoretical aspects of antibiotic diffusion into microbial biofilms. Antimicrob Agents Ch.

[19] Gilbert P, Maira-Litran T, McBain AJ (2002). The physiology and collective recalcitrance of microbial biofilm communities. Adv Microb Physiol.

[20] Macià MD, Rojo-Molinero E, Oliver A (2014). Antimicrobial susceptibility testing in biofilm-growing bacteria. Clin Microbiol Infect.

[21] Neu TR, Lawrence JR (2014). Investigation of microbial biofilm structure by laser scanning microscopy. Adv Biochem Eng Biot.

[22] Wright CJ, Shah MK, Powell LC (2010). Application of AFM from microbial cell to biofilm. Scanning.

[23] Mah TF, O'Toole GA (2001). Mechanisms of biofilm resistance to antimicrobial agents. Trends Microbiol.

[24] Ceri H, Olson ME, Stremick C (1999). The Calgary Biofilm Device: new technology for rapid determination of antibiotic susceptibilities of bacterial biofilms. J Clin Microbiol.

[25] Kostakioti M, Hadjifrangiskou M, Hultgren SJ (2013). Bacterial biofilms: development, dispersal, and therapeutic strategies in the dawn of the postantibiotic era. CSH Perspect Med.

[26] Toutain CM, Caizza NC, Zegans ME (2007). Roles for flagellar stators in biofilm formation by *Pseudomonas aeruginosa*. Res Microbiol.

[27] Pratt LA, Kolter R (1998). Genetic analysis of *Escherichia*
*coli* biofilm formation: roles of flagella, motility, chemotaxis and type I pili. Mol Microbiol.

[28] Kwasny SM, Opperman TJ (2010). Static biofilm cultures of Gram-positive pathogens grown in a microtiter format used for anti-biofilm drug discovery. Curr Protoc Pharmacol.

[29] Moskowitz SM, Foster JM, Emerson J (2004). Clinically feasible biofilm susceptibility assay for isolates of *Pseudomonas*
*aeruginosa* from patients with cystic fibrosis. J Clin Microbiol.

[30] Bhattacharya M, Wozniak DJ, Stoodley P (2015). Prevention and treatment of *Staphylococcus aureus* biofilms. Expert Rev Anti-Infe.

[31] Francolini I, Donelli G (2010). Prevention and control of biofilm-based medical-device-related infections. FEMS Immunol Med Mic.

[32] Doring G, Hoiby N (2004). Early intervention and prevention of lung disease in cystic fibrosis: a European consensus. J Cyst Fibros.

[33] Fernández-Olmos A, García-Castillo M, Maiz L (2012). In vitro prevention of *Pseudomonas*
*aeruginosa* early biofilm formation with antibiotics used in cystic fibrosis patients. Int J Antimicrob Ag.

[34] Sans-Serramitjana E, Jorba M, Fusté E (2017). Free and nanoencapsulated tobramycin: effects on planktonic and biofilm forms of *Pseudomonas*. Microorganisms.

[35] Zhou YF, Shi W, Yu Y (2015). Pharmacokinetic/Pharmacodynamic correlation of cefquinome against experimental catheter-associated biofilm infection due to *Staphylococcus*
*aureus*. Front Microbiol.

[36] Moormeier DE, Bayles KW (2017). *Staphylococcus aureus* biofilm: a complex developmental organism. Mol Microbiol.

[37] Sans-Serramitjana E, Jorba M, Pedraz JL (2017). Determination of the spatiotemporal dependence of *Pseudomonas aeruginosa* biofilm viability after treatment with NLC-colistin. Int J Nanomed.

[38] Quilès F, Saadi S, Francius G (2016). In situ and real time investigation of the evolution of a *Pseudomonas fluorescens* nascent biofilm in the presence of an antimicrobial peptide. BBA-Biomembranes.

[39] Pamp SJ, Gjermansen M, Johansen HK (2008). Tolerance to the antimicrobial peptide colistin in *Pseudomonas aeruginosa* biofilms is linked to metabolically active cells, and depends on the pmr and mexAB-oprM genes. Mol Microbiol.

[40] Hengzhuang W, Ciofu O, Yang L (2013). High β-lactamase levels change the pharmacodynamics of β-lactam antibiotics in *Pseudomonas aeruginosa* biofilms. Antimicrob Agents Ch.

[41] (2001). Molecular probes, Product information LIVE/DEAD ® BacLight™ bacterial viability Kit.

[42] Binnig G, Quate CF, Gerber C (1986). Atomic force microscope. Phys Rev Lett.

[43] Li A, Ho B, Ding JL (2010). Use of atomic force microscopy as a tool to understand the action of antimicrobial peptides on bacteria. Method Mol Biol.

[44] López-Jiménez L, Arnabat-Domínguez J, Viñas M (2015). Atomic force microscopy visualization of injuries in *Enterococcus*
*faecalis* surface caused by Er,Cr:YSGG and diode lasers. Med Oral Patol Oral Cir Bucal.

[45] Zalacain A, Merlos A, Planell E (2017). Clinical laser treatment of toenail onychomycoses. Laser Med Sci.

[46] Gonçalves S, Silva PM, Felício MR (2017). Psd1 effects on *Candida albicans* planktonic cells and biofilms. Front Cell Infect Mi.

[47] Mularski A, Wilksch JJ, Wang H (2015). Atomic force microscopy reveals the mechanobiology of lytic peptide action on bacteria. Langmuir.

[48] Ramalingam B, Parandhaman T, Das SK (2016). Antibacterial effects of biosynthesized silver nanoparticles on surface ultrastructure and nanomechanical properties of Gram-negative bacteria viz. *Escherichia*
*coli* and *Pseudomonas*
*aeruginosa*. ACS Appl Mater Inter.

[49] Canetta E, Riches A, Borger E (2014). Discrimination of bladder cancer cells from normal urothelial cells with high specificity and sensitivity: combined application of atomic force microscopy and modulated Raman spectroscopy. Acta Biomater.

[50] Henderson JM, Waring AJ, Separovic F (2016). Antimicrobial peptides share a common interaction driven by membrane line tension reduction. Biophys J.

[51] Angélique L, Frederik W, Garmi J (2015). The potential use of natural and structural analogues of antimicrobial peptides in the fight against neglected tropical diseases. Molecules.

[52] Vinuesa T, Herráez R, Oliver L (2017). Benznidazole nanoformulates: a chance to improve therapeutics for chagas disease. Am J Trop Med Hyg.

[53] Luque-Ortega JR, Rivas L (2010). Characterization of the leishmanicidal activity of antimicrobial peptides. Method Mol Biol.

[54] Pinto EG, Pimenta DC, Antoniazzi MM (2013). Antimicrobial peptides isolated from *Phyllomedusa*
*nordestina* (Amphibia) alter the permeability of plasma membrane of *Leishmania* and *Trypanosoma*
*cruzi*. Exp Parasitol.

[55] Tejería A, Pérez-Pertejo Y, Reguera RM (2016). Antileishmanial effect of new indeno-1,5-naphthyridines, selective inhibitors of *Leishmania*
*infantum* type IB DNA topoisomerase. Eur J Med Chem.

[56] Malucelli MI, Niero R, Lucchiari PH (1995). Evaluation of the polarographic technique for assay of the viability of freeze-dried BCG vaccine: II. Viability of the vaccine assessed by polarography, Warburg respirometry and colony counting. Vaccine.

[57] Wayne PA (2002). Reference method for broth dilution antifungal susceptibility testing of yeasts.

[58] John HR (2008). Reference method for broth dilution antifungal susceptibility testing of filamentous fungi.

[59] (2017). EUCAST method for susceptibility testing of yeasts (version 7.3.1). http://www.eucast.org/ast_of_fungi/methodsinantifungalsusceptibilitytesting/susceptibility_testing_of_yeasts/.

[60] (2017). EUCAST method for susceptibility testing of moulds (version 9.3.1). http://www.eucast.org/ast_of_fungi/methodsinantifungalsusceptibilitytesting/susceptibility_testing_of_moulds/.

[61] Simitsopoulou M, Chatzimoschou A, Roilides E (2016). Candida species: methods and protocols, methods in molecular biology. Method Mol Biol.

[62] Vale-Silva LA, Buchta V (2006). Antifungal susceptibility testing by flow cytometry: is it the future?. Mycoses.

